# Feasibility, acceptance and effects of pulsed magnetic field therapy in patients with post-COVID-19 fatigue syndrome: A randomized controlled pilot study

**DOI:** 10.1007/s00508-025-02522-w

**Published:** 2025-03-17

**Authors:** Mohammad Keilani, Margarete Steiner, Julia Sternik, Jim Schmeckenbecher, Ralf Harun Zwick, Barbara Wagner, Richard Crevenna

**Affiliations:** 1 Department of Physical Medicine, Rehabilitation and Occupational Medicine, Medical University of Vienna, Währinger Gürtel 18–20, 1090 Vienna, Austria; 2 Therme Wien Med, Ludwig Boltzmann Institute for Rehabilitation Research, Vienna, Austria

**Keywords:** Post-COVID-19, Fatigue, Pulsed magnetic field therapy, Acceptance, Feasibility

## Abstract

**Introduction:**

The aim of this randomized, single-blinded, placebo-controlled pilot study was to explore the feasibility, acceptance and effects of pulsed electromagnetic field therapy (PEMF) in patients with post-COVID-19 fatigue syndrome.

**Methods:**

A total of 20 patients were included in the study. They were randomly assigned to an intervention group (*n* = 10, male:female = 4 : 5, age = 45 ± 9 years) and a placebo group (*n* = 10, m:f = 4:6, age: 39 ± 23 years).

A Papimi™ Delta professional electromagnetic field therapy device was used for treatment. Controls received a placebo PEMF. In total 10 PEMF sessions (30 min., 2× per week) were applied.

Physical performance, health-related quality of life, fatigue, work ability, sleep, pain, anxiety and depression were assessed at baseline, posttreatment and at follow-up 5 weeks after treatment. Feasibility and acceptance were assessed posttreatment.

**Results:**

The intervention group showed a notable improvement in the 6 minutes walking test. The use of PEMF improved fatigue (measured with the Brief Fatigue Inventory and the Multidimensional Fatigue Inventory). Also, the depression subscale of the Hospital Anxiety and Depression Scale, the Insomnia Severity Index, the Work Ability Index and most subscales of the Short Form (SF) 36 questionnaire showed improvement.

From the placebo group three patients dropped out. There were no drop-outs in the intervention group. In the intervention group, PEMF was well-accepted and patients showed very good adherence.

**Conclusion:**

The results of this pilot study indicate that PEMF is feasible and well accepted. Furthermore, this study population showed improvements in physical and mental health in the intervention group. This study is a promising contribution to this growing research field and provides the required evidence for future efficacy studies on PEMF for post-COVID-19 patients.

## Introduction

Post-COVID-19 syndrome (PCS) describes a persistent complex of symptoms following SARS-CoV-2 infection [1, 2]. A common symptom in PCS is fatigue. It usually worsens after physical or mental exertion (post-exertional malaise). Fatigue is often associated with a deterioration in health-related quality of life (HRQOL) including functional impairment, sleep disturbances, exacerbations of pain and decline in cognitive functions, particularly in memory and higher order functions [1, 2].

Mitochondrial dysfunction in individuals suffering from long-COVID has been reported in the literature [3], suggesting that impaired mitochondrial energy production may be a possible cause of fatigue symptoms [4, 5].

Pulsed electromagnetic field therapy (PEMF) might modulate mitochondrial dynamics and play a role in regulating mitochondrial function [6, 7]. For example, in a recent study Yang et al. concluded that exposure to PEMF might accelerate angiogenesis in human umbilical vein endothelial cells, probably by inducing reprogramming of energy metabolism and mitochondrial fission [7]; however, the exact mode of action of electromagnetic fields is still unknown.

Clinical studies on the effects of PEMF on PCS-related fatigue are rare. Previously, a case report was published by Wagner et al. indicating that fatigue, work ability, HRQOL and psychological well-being improved over the course of the treatment and showed stable results 6 weeks later [8].

Based on these findings, a randomized, controlled pilot study was performed to explore the effects of PEMF in patients suffering from post-COVID-19-related fatigue.

## Methods

### Setting

This randomized, parallel single-blinded placebo-controlled randomized control trial with a 1:1 patient:control group ratio. This exploratory pilot study took place at the Department of Physical Medicine, Rehabilitation and Occupational Medicine, Medical University of Vienna, Austria and was approved by the Ethics Committee of the Medical University of Vienna (EK-Nr: 1764/2021). Written informed consent was obtained from all patients prior to enrolment. The patients were blinded to the intervention (single-blind design). The treating staff were not blinded to the allocation because we used different spool applicators, which had to be changed during the therapy sessions.

### Inclusion criteria

- Individuals who have experienced COVID-19 disease and are suffering from post-COVID-19 fatigue syndrome, with a score of 2–4 on the Post Covid Functional Status Scale (PCFS) at baseline. The PCFS is used as a measure to assess the consequences of COVID-19 and its impact on functional status. It has been designed to cover the entire range of functional limitations from: grade 0 = no functional limitations to grade 4 = severe functional limitations and grade 5 = death [9].
- Age: 18–65 years.
- Consent to participate in the study.

### Exclusion criteria

- Contraindications to PEMF therapy: electronic implants (e.g. implantable cardioverter defibrillator, pacemaker, cochlear implants), pregnancy and ring-shaped metals in the body.
- Poor general health condition that does not allow participation in the study.
- Other therapy for fatigue syndrome during the study (physical therapies, medication).
- Acute COVID-19 disease, positive SARS-CoV 2 test results.
- Relevant psychiatric disorders or cognitive impairments.
- Language barrier.
- If regular participation in the therapy/study is not possible due to time, personal, physical or other reasons.
- Lack of consent.

### Patients

Recruitment started on 5 July 2023 and ended on 8 July 2024. Follow-up assessments were completed on 3 September 2024. Patients or employees of the University Hospital Vienna and the Medical University of Vienna who are referred to our department or had a suspected post-COVID-19 diagnosis were screened.

**Fig. 1:**
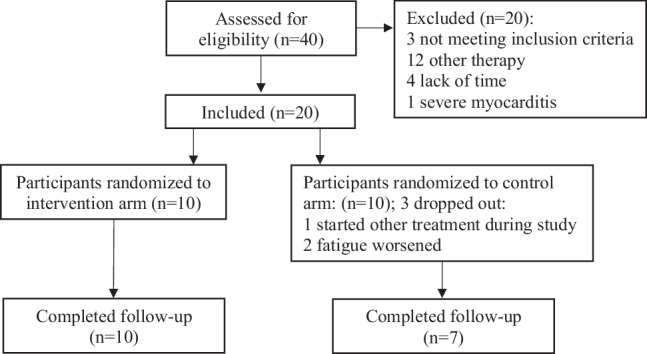
Patient recruitment flow diagram

A total of 40 individuals were assessed for eligibility and 20 patients were excluded according to the predefined exclusion criteria (Fig. 1).

In all, 20 patients were included in the present study. The participants were randomized into an intervention group and a placebo group by using simple randomization [9]. Randomization algorithms were created using R (R Foundation, Indianapolis, Indiana, United States) by an external statistician, research staff at the clinic were then provided with concealed and sealed envelopes that contained the allocation of participants to either control or intervention groups. Allocation of participants took place after they agreed to participate in the study. The participants were blinded to the intervention.

Demographic and clinical data of included patients are presented in Table 1.

**Table 1:** Demographic and clinical data of the study population at baseline (T0)

Variable	Control group (*n* = 10)	Intervention group (*n* = 10)
–	Median (IQR)	
Age (years)	39.2 (23.33)	44.51 (8.65)
Weight (kg)	73.8 (9.75)	77.2 (30)
Height (cm)	179 (8.25)	175 (11.75)
Body mass index	23.51 (3.26)	26.74 (7.81)
Time from diagnosis to treatment (days)	277.5 (183)	278 (205.75)
Post-COVID-19 Functional Status Scale	3 (0)	3 (0)
–	*N*	
Sex—female/male	6/4	5/5
Partnership—yes	5	6
Smoker—yes	0	0
Ex-smoker—yes	2	3
High school and higher	8	9
*Comorbidities*^a^		
Musculoskeletal disease	3	2
Hypertension	2	3
Cardiovascular diseases	0	1
Gastrointestinal diseases	3	1
Thyroid gland diseases	2	1
Pulmonary diseases	0	2
Adiposity	0	1
Others	1	0
*History of completed post-COVID-19 treatment*		
Physiotherapy—yes	5	5
Strength training—yes	5	4
Endurance training—yes	4	5
Psychotherapy—yes	3	2

*IQR* interquartile range

^a^All comorbidities were mild and did not lead to exclusion from the study.

### Intervention

In the present study, the Papimi™ Delta professional electromagnetic field therapy device (Pulse Dynamics Ltd., Pipinou 1 & Souliou, 17342 Agios Dimitrios, Greece) was used for treatment [10].

The PEMF is based on the principle of ion induction. The PEMF pulse is like a damped oscillation with a short pulse duration of ~50 µs. The basic frequency is ~240 kHz. In the maxima and minima of the damped oscillation, high-frequency oscillation peaks in the megahertz to gigahertz range arise. The pulse rate can be varied between 1 and 8 Hz. High voltages (up to 40 kV) and peak currents (up to 10 kA) arise in the applicator spool. As a result, the Papimi™ device achieves delivery of energy per pulse of about 96 Watt (Joule) with a magnetic flux density of 50–100 mT. The device allows a penetration of the body tissue depth of about 20 cm [10].

The patients received 10 sessions of PEMF twice weekly for 5 weeks. Each session lasted 30 min.

The application sites were selected following the device manufacturer’s manual [10]. Therapy was applied to the epigastric area for 9 min and 3 min over the sternum. Then it was applied to the lower back and over the lungs for 9 min (dorsal area), to the soles of both feet for 3 min and to the pelvic floor area for 6 min.

A large spool applicator (diameter 20 cm) was used in all areas, except for the pelvic floor, where we used a smaller one (diameter 15 cm). The pulse rate was 2.5 Hz in the dorsal area and 1 Hz for all other locations.

For all positions 100% of maximum power (about 96 W) was chosen, except for the pelvic floor when 75% of maximum power was used. Furthermore, we adapted the intensity individually by inserting felt mats (provided by the manufacturer).

For the control group, optically identical sham applicators were used that sound like real magnetic field applicators and cause a mechanical vibration but do not generate a magnetic field.

### Assessment

Demographic and clinical data of all participants were collected at baseline. Feasibility and acceptance were assessed after the treatment. The assessment of feasibility focused on the drop-out rate and adverse events. The assessment of acceptance focused on participation rates. Furthermore, the patients were asked if the treatment “was perceived as effective” (response options: yes/no/no answer).

Validated clinician-reported outcomes (CROMS) for objective assessment of physical performance as well as questionnaires were used for assessment before treatment (T0), after 5 weeks of treatment (T1) and 5 weeks after the end of treatment (T2).

As post-COVID can lead to increased fatigue and decreased exercise capacity the 6 minute walking test (6 MWT) was used for objective assessment [1, 2]. It is an easy, simple and inexpensive test, which is widely used in clinical practice. It provides information on submaximal (in some cases, maximal) exercise capacity in patients with cardiovascular and respiratory diseases [11]. The outcome is the maximum distance that a patient can cover after walking for 6 minutes. It is a useful test in post-COVID-19 follow-up as it correlates with the severity of acute disease and with functional impairment caused by PCS [12].

Furthermore, we assessed the 30 s sit to stand test (30 s STS). The 30 s STS can be valuable for assessing both lower limb muscle endurance and functional capacity, making it useful for quantifying submaximal exercise capacity, particularly in home health settings.

It is an easy and quick method in which patients are instructed to stand up and sit down on a chair with their arms folded across their chest as many times as possible for 30 s. This test can indicate global muscle wasting. It is used to assess lower limb muscle power which is related to physical performance [13].

Hand grip strength reflects upper extremity strength and is used as an indicator of health and functionality. [14, 15]. Therefore, we measured the hand grip strength of the dominant hand using a Jamar® dynamometer (Patterson Medical, Warrenville, Illinois, United States of America) [14]. Patients were examined in a standardized position. During the measurement they were seated with the elbow bent at a right angle beside the body and in a neutral wrist position. The position of the dynamometer handle was chosen according to individual hand size [14, 15] and three maximum voluntary grip strength contractions were performed with the right and left hand in alternating order. Maximum hand grip strength was measured in pounds (lbs) [15].

Self-reporting validated questionnaires were used to assess the effect of PEMF treatment on functional status, fatigue, pain, depression and anxiety, insomnia, general HRQOL and work ability.

Fatigue, which is obviously the most important parameter, was assessed by using the Brief Fatigue Inventory (BFI) as well as the Multidimensional Fatigue Inventory (MFI).

The BFI is a screening questionnaire designed to assess severity and impact of fatigue on daily functioning (activity, mood, walking ability, work, relationships with other people and enjoyment of life) in the past 24 h (10 items) [16, 17]. The MFI is a 20-item self-report instrument designed to measure fatigue. It covers the following dimensions: general fatigue, physical fatigue, reduced activity, reduced motivation and mental fatigue [18, 19].

The symptom pain was assessed by using the PainDETECT questionnaire (PD-Q).

The PainDETECT questionnaire was developed as screening tool to identify neuropathic pain. It is used across various clinical settings, including conditions such as osteoarthritis, low back pain, and fibromyalgia. The questionnaire assesses pain intensity, pain pattern and pain quality [20]. We used the 11-point numerical rating scale to measure current pain.

Depression and/or anxiety is frequent in post-COVID-19 fatigue syndrome [1, 2]. We used the Hospital Anxiety and Depression Scale (HADS) for assessment which is a screening tool to determine the severity of anxiety and depression in patients with a somatic disease. The focus of this questionnaire is solely on psychological symptoms of anxiety and depression to avoid confounders due to somatic comorbidities. It also captures milder forms of anxiety and depression, which are common in somatic illnesses (14 items). The total score of the two subscales, “anxiety” and “depression” can be used as a measure of overall psychological distress [21].

Furthermore, insomnia is a common symptom associated with post-COVID-19 fatigue syndrome. The Insomnia Severity Index (ISI) was used which is a brief and valid tool to quantify the severity of insomnia symptoms, frequently used in both research and clinical settings (7 items) [22].

Fatigue is often associated with a deterioration in HRQOL. The Short Form Health Survey is a questionnaire to measure HRQOL across multiple diseases. It offers a number of strong practical advantages for examining the HRQOL [23, 24]. Its strengths include its applicability to many disease groups as well as to the general population, allowing it to be used for comparisons between different diseased as well as healthy populations. It covers eight dimensions: physical functioning (PF), role physical (RP), bodily pain (BP), general health (GH), vitality (VT), social functioning (SF), role emotional (RE) and mental health (MH), conceptually divided into “physical component summary (PCS)” and “mental component summary (MCS)” as well as health changes [23, 24].

Many post-COVID-19 patients experience a prolonged recovery period, resulting in temporary or long-term work limitations [1, 2]. Therefore, we also assessed work ability. The Work Ability Index (WAI) short version evaluates work ability in relation to current physical and psychological work demands [25]. The WAI assesses individual work capacity concerning current work conditions, covering subjective assessment of physical and psychological work demands, work limitations due to illness, sick leave in the previous year and a predictive evaluation of the work ability for the following 2 years. The WAI includes 10 items grouped into 7 dimensions. The total WAI score reflects the individual’s self-assessed ability to manage current work requirements [25].

### Statistics

In order to conduct an intention-to-treat analysis, data were imputed by group using multiple imputation, with the mice package being employed for this purpose [26]. Subsequently, the data were subjected to descriptive statistical analysis. For variables exhibiting non-normal distributions, medians and interquartile ranges were employed, whereas for variables with normal distributions, means and standard deviations were utilized. Given the limited sample size, we elected to refrain from employing inferential statistics, as the resulting *p*-values would have been unreliable due to the limited statistical power supporting the analysis.

## Results

During the study period, the condition of three patients from the control group worsened due to fatigue, they discontinued therapy and therefore dropped out. There was no follow-up or end of the study visit in the three drop-outs of the control group, because all three patients worsened remarkably. Therefore, they were not able to take part in the follow-up examination.

In the intervention group, no patients dropped out, moreover only mild symptoms were reported after the first therapy session. More specifically, three patients were tired, two patients reported that they had headache, and one patient had muscle soreness. In the control group, two patients reported tiredness, one patient had vertigo after the first treatment and one patient sometimes had headache after the therapy sessions. The PEMF has been shown to be safe, as no other adverse events were reported.

In the intervention group 5 therapy sessions out of 100 were missed due to an infection and the remaining therapies were carried out in other appointments. In the control group, 7 (out of 10 patients completed the therapy). These patients missed 7 (out of 70) therapy sessions and had to make up for it.

Out of 10 patients of the intervention group 7 perceived the treatment as effective, 2 patients perceived the treatment as not effective and 1 patient chose the answer option “no response”. In the control group, only two (out of seven) patients perceived the treatment as effective, three patients perceived the treatment as not effective and two patients selected the answer option “no response”.

The results of the 6 MWT (pre-post, follow-up assessment at T1: +10% vs. 11%, and T2: +14% vs. 9%) notably improved in the intervention group more than in the control group at T2 (Table 2).

**Table 2:** Descriptive results of PEMF-treatment in the assessed patients

–	***Non-normally distributed variables***					
–	**Baseline (T0)**		**After 5 weeks of treatment (T1)**		**Follow-up 5 weeks after treatment (T2)**	
*Variable name*	*Control Median (IQR)*	*Intervention Median (IQR)*	*Control Median (IQR)*	*Intervention Median (IQR)*	*Control Median (IQR)*	*Intervention Median (IQR)*
PCFS	3 (0)	3 (0)	3 (0.75)	2 (1)	2 (0.75)	2 (0)
30 s STS	10.5 (7.25)	14.5 (4)	14 (4.75)	19 (6.5)	13.5 (5.75)	18.5 (7.25)
MFI General Fatigue	17.5 (3.25)	17 (2.75)	17.5 (4)	14 (7)	17 (3)	12 (4.75)
MFI Physical Fatigue	17.5 (3)	18 (7.25)	17 (1.75)	15.5 (8.5)	15.5 (4.75)	12 (8.25)
MFI Reduced Activity	17.5 (3)	16.5 (6)	16.5 (1.75)	14 (9.75)	16 (0)	11 (2.75)
MFI Reduced Motivation	11 (5)	10 (6.5)	10 (8)	8.5 (6.75)	7 (3)	8 (5)
MFI Mental Fatigue	15 (2.75)	15.5 (5.75)	16.5 (4)	15 (12.75)	14 (4.5)	12 (8)
HADS A	8.5 (4.5)	6 (3.75)	5 (8.25)	5 (1.75)	5.5 (12)	4.5 (1)
HADS D	7 (6.75)	6.5 (8.5)	6 (5.25)	4 (6.25)	6 (3)	3 (6.75)
PD‑Q	3 (2.75)	1.5 (3.5)	4 (2.75)	1 (2.75)	3.5 (1.75)	2 (1.75)
SF-36 Change in health status	3.5 (1.75)	3 (0)	3 (0.75)	3 (1)	3 (1.5)	3 (0.75)
SF-36 PF	32.5 (10)	55 (33.75)	30 (10)	67.5 (41.25)	40 (36.25)	70 (25)
SF-36 RP	0 (0)	0 (0)	25 (25)	12.5 (68.75)	0 (0)	50 (66.67)
SF-36 BP	62 (56.5)	62 (50.25)	51 (22.25)	67 (19.5)	51 (21)	73 (18.5)
SF-36 GH	27.5 (21.25)	27.5 (13.75)	25 (22.5)	35 (11.75)	22.5 (10)	40 (34.25)
SF-36 VT	15 (10)	17.5 (22.5)	17.5 (5)	37.5 (51.25)	25 (8.75)	40 (31.25)
SF-36 SF	25 (59.38)	31.25 (53.12)	43.75 (12.5)	62.5 (53.12)	50 (31.25)	68.75 (21.88)
SF-36 RE	100 (91.67)	16.66 (100)	33.33 (91.67)	83.34 (91.67)	33.33 (83.34)	100 (25)
SF-36 MH	50 (21)	62 (24)	68 (31)	70 (35)	56 (40)	72 (24)
SF-36 MCS	43.34 (15.14)	35.56 (13.26)	42.9 (7.29)	45.46 (26.48)	35.61 (11.6)	48.6 (16.29)
WAI	17 (9)	23.75 (17.88)	15 (8.5)	26 (10.38)	17 (11.5)	28.25 (13.75)
–	***Normally distributed variables***					
–	**Baseline (T0)**		**After 5 weeks of treatment (T1)**		**Follow-up 5 weeks after treatment (T2)**	
*Variable name*	*Control M (SD)*	*Intervention M (SD)*	*Control M (SD)*	*Intervention M (SD)*	*Control M (SD)*	*Intervention M (SD)*
6 MWT	448.8 (214.09)	549.7 (92.53)	199.1 (163.)	605.1 (74.67)	488.8 (139.41)	628.7 (80.59)
HGS dominant (lbs)	66.9 (25.48)	81 (31.23)	71.8 (21.63)	88.1 (29.46)	74.1 (28.6)	87 (21.96)
BFI	6.12 (1.55)	5.16 (1.39)	5.29 (1.9)	4.26 (2.42)	4.38 (1.57)	2.88 (1.59)
MFI total	74.8 (9.22)	72.9 (13.7)	72.6 (12.83)	61.6 (23.31)	70.7 (9.98)	52.4 (19.77)
ISI	17.1 (7.23)	11.6 (3.44)	15.1 (5.8)	7.8 (2.97)	15.5 (3.54)	7.2 (4.66)
HADS total score	17.5 (9.73)	14.6 (6.6)	14.6 (7.73)	11.1 (7.42)	15 (7.33)	8.6 (4.81)
SF-36 PCS	29.99 (7.46)	33.23 (12.73)	31.86 (7.67)	38.55 (11.06)	34.33 (8)	42.58 (9.85)

*PCFS* Post COVID-19 Functional Status Scale, *30* *s STS* 30 second sit-to-stand test, *MFI* Multidimensional Fatigue Inventory, *HADS* Hospital Anxiety and Depression Scale, *HADS A* anxiety, *HADS D* depression, *PD‑Q current pain* PainDETECT questionnaire (11-point numerical rating scales to measure current pain), *SF-36* Short Form Health Survey (SF-36), *FS-36 * change in health status, *FS-36 PF* physical functioning, *SF-36 RP* role physical, *SF-36 BP* bodily pain, *SF-36 GH* general health, *SF-36 VT* vitality, *SF-36 SF* social functioning, *SF-36 RE* role emotional, *SF-36 MH* mental health, *MHC* mental component summary, *PCS* physical component summary, *WAI* Work Ability Index, *6 MWT* Six Minute Walking Test, *HGS dominant* handgrip strength of the dominant hand, *BFI* Brief Fatigue Inventory, *ISI* Insomnia Severity Index

There was no notable difference in the 30 s STS (T1: +31% vs. +33%, T2: +27% vs. 28%) as well as hand grip strength (T1: +9% vs. 7%, T2: +7% vs. +11%) (Table 2).

There was an improvement of BFI at T1 (−17% vs. −14%) as well as T2 (−44% vs. −28%) for the intervention group, compared to the control group (Table 2).

Furthermore, the MFI total score (−26% vs. −4%) as well as the MFI subscales “physical fatigue” (−33% vs. −11%) and “reduced activity” (−33% vs. −8%) notably improved at T2 after real PEMF compared to the control group (Table 2).

An improvement in sleep quality (measured with ISI) was already observed at T1, that was higher in the intervention group than in the control group (T1: −33% vs. −12%, T2: −38% vs. −10%) (Table 2).

The HRQOL (SF-36) improved for the intervention group in the “physical component summary” (T1: +16% vs. +6%, T2: +28% vs. 14%) as well as in the “mental component summary” (T1: +28% vs. −1%, T2: +36% vs. −18%). The SF-36 “change in health status” score showed no change for the intervention group but a slight improvement for the control group over time (T1: 0% vs. −1%, T2: 0% vs. −1%) (Table 2).

Furthermore, there was an improvement for the intervention group in the SF-36 subscales “physical functioning” (T1: +22% vs. −8%, T2: +27% vs. +23%), “role physical” (T2: +50% vs. ± 0%), “bodily pain” (T1: +8% vs. −18%, T2: +18% vs. −18%) “general heath perceptions” (T1: +27% vs. −9%, T2: +45% vs. −8%), “vitality” (T1: +114% vs. 16%, T2: +128% vs. +66%), “social functioning” (T1: +100% vs. +75%, T2: +120% vs. +100%) and “role emotional” (T1: +400% vs. −77%, T2: +500% vs. −77%), compared with the control group (Table 2).

The intervention group improved less in T1 concerning “role physical” (T1: +12.5% vs. +25%), and “mental health” (T1: +12% vs. +36%) (Table 2).

There was an improvement of self-reported work ability (measured by the use of the WAI) at T1 (+10% vs. −12%) as well as T2 (+19% vs. ± 0%) for real PEMF (Table 2).

Current pain (measured with the PD-Q) notably improved at T1 in the intervention group but there was no clinically notable group difference at T2 (T1: −33% vs. +33%, T2: +13% vs. +11%) (Table 2).

The HADS anxiety subscale (T1: −17% vs. −42%, T2: −25% vs. −36%) improved less in the intervention group, whereas the HADS depression subscale showed more improvement for the intervention group (T1: −39% vs. −14%, T2: −54% vs. −14%) (Table 2). The HADS total score improved for the intervention group more than in the control group (T1: −34% vs. −17%, T2: −53% vs. −16%) (Table 2).

## Discussion

The PEMF can be applied to treat a variety of disorders. The advantages of PEMF seem to be that the intervention is noninvasive and no serious side effects, such as post-exertional malaise are known. Growing evidence from clinical studies and clinical routine suggests that ion induction therapy may be effective for osteoarthritis, inflammation and accelerated regeneration. It has also been shown to enhance bone fracture healing and alleviate low back pain [27–33].

In the present study, the Papimi™ Delta professional electromagnetic field therapy device (Pulse Dynamics Ltd., Pipinou 1 & Souliou, 17342 Agios Dimitrios, Greece) was used for treatment [10]. Pulsed electromagnetic field therapy (PEMF) is used to treat different pathologies of bones, muscles and joints [27–33]. It provides a cost-effective and safe therapeutic modality with growing popularity and use in physical medicine and rehabilitation. We daily use PEMF in common clinical practice for several musculoskeletal pain disorders.

In patients suffering from fatigue, PEMF treatment has been applied in patients with multiple sclerosis with mixed success [34, 35]; however, previous studies on multiple sclerosis-associated fatigue were performed using heterogeneous treatment parameters.

The scientific literature on magnetic field therapy for COVID-19 and post-COVID-19 fatigue is still rare.

In a study of patients with COVID-19 pneumonia, low-frequency PEMF therapy was successfully used to reduce respiratory symptoms, pain, anxiety and depression, and improve quality of life [36].

Zhang et al. recently performed a case report on transcranial magnetic stimulation using the electromagnetic brain pulse technique (frequency: 9–15 Hz, flux density: 0.6 T) in a patient with long COVID. After 10 therapy sessions, the patient had improvements in mood, sense of smell, and brain fogging [37].

In a further case report by Schaefer et al., PEMF (frequency of 550 Hz, flux density: 1 mT) was applied once at the area of the stellate ganglion [39]. Immediately post-treatment isometric muscle strength of hip flexors and elbow flexors improved and the subjective long COVID symptoms resolved the following day. At 6 months the patient’s long COVID symptoms have not returned [38].

In 2022 a case report on the use of PEMF for post-COVID-19-related fatigue was published by our working group [8]. After 5 weeks of PEMF, notable improvements were observed in fatigue, work ability, HRQOL and mental well-being. The positive results were maintained 6 weeks post-treatment.

The three reported case reports used very heterogeneous therapy protocols. Therefore, they cannot be compared to each other [8, 37, 38].

The present study aimed to describe the feasibility and acceptance of an intervention by using PEMF with the goal to improve symptoms related to post-COVID-19 fatigue syndrome.

The results showed that PEMF was both feasible and well accepted. Patients in the intervention group showed excellent compliance and adherence, with only 5% of 100 sessions missed in the intervention group, compared to 10% of 70 sessions missed in the placebo group. Additionally, there were three drop-outs in the placebo group due to increased fatigue, whereas no participants dropped out of the intervention group, indicating that real PEMF was well tolerated. Moreover, no patients in the intervention group experienced worsening of their condition.

Additionally, this intervention successfully reduced fatigue, improved physical performance and work ability, sleep quality and enhanced most aspects of HRQOL in the intervention group (Table 2).

The benefits of PEMF therapy extended beyond the initial treatment, with sustained improvements observed in physical activities, mental health, sleep quality, overall quality of life and work ability (Table 2). These results seem to be in accordance with the theory that PEMF (as with other physical stimuli) might enhance tissue regeneration [27, 32]. Furthermore, repetitive exposure to PEMF might notably improve mitochondrial function, which has been damaged by hyperinflammation [37, 39].

The intervention group improved less in T1 concerning “role physical” and “mental health” but at T2, both parameters notably changed in favor of the intervention group.

There was no difference between the groups in the 30 s STS and hand grip strength, while the 6 MWT results demonstrated significant improvements favoring the intervention group. These findings suggest that PEMF may impact aerobic capacity rather than muscle strength, potentially supporting the idea that PEMF can enhance mitochondrial function [39].

The minimal clinically important difference (MCID) is defined as the smallest change in score that patients perceive as beneficial [40].

For a number of outcomes presented in this paper, there is no agreed MCID but we only know that a certain direction is preferable (e.g., it is preferable to be able to walk further). Therefore, we believe that it is scientifically sound to add MCIDs and binary values based on these MCIDs only when there is an overwhelming scientific consensus.

The available literature with respect to rehabilitation of patients with post-COVID-19 suggests a MCID for 6 MWT of more than 35 m for adult patients with cardiopulmonary diseases [40]. In our study, the 6 MWT improved for the intervention group at T1 by 55 m and at T2 by 79 m. The placebo group worsened at T1 by 246m and improved at T2 by 40 m. These results indicate a clinical benefit after PEMF [40].

Furthermore, the MFI total score improved notably at T2 after real PEMF by 20.5 units. The MCID for the MFI total score was suggested to be −14.3 units [40].

Unfortunately, for the remaining parameters there are no data with respect to MCID in patients suffering from post-COVID-19 fatigue syndrome.

The HADS anxiety subscale and current pain (assessed with the numeric rating scale of the PD-Q) showed no benefit for the intervention group. These results suggest that PEMF might not have a measurable short-term effect on these parameters in patients suffering from post-COVID-19.

In summary, PEMF seems to be an effective approach, which can be embedded into the comprehensive multimodal and interdisciplinary rehabilitation concept of certain patients suffering from post-COVID-19 fatigue syndrome [41, 42].

The present study has several limitations. This was a pilot study with a limited statistical power showing the changes in a descriptive matter. Nevertheless, this was the first randomized, placebo-controlled trial, in which feasibility and acceptance as well as the effectiveness of PEMF for post-COVID-19 fatigue syndrome was demonstrated.

## Conclusion

The results of this pilot study indicate safe feasibility and good acceptance of PEMF for patients with post-COVID-19 fatigue syndrome. Furthermore, this study population showed improvements in physical and mental health in the intervention group. This study seems to be an important contribution to this growing research field and provides the required evidence for future larger randomized controlled efficacy studies on PEMF in post-COVID-19 patients.

Nevertheless, further research including high-quality randomized controlled clinical trials should focus on effects of PEMF on patients with post-COVID-19 fatigue syndrome.

## References

[1] Ely EW, Brown LM, Fineberg HV. Long Covid defined. N Engl J Med. 2024; 10.1056/NEJMsb2408466. 39083764 10.1056/NEJMsb2408466 PMC11687645

[2] Greenhalgh T, Sivan M, Perlowski A, Nikolich JŽ. Long COVID: a clinical update. Lancet. 2024;404(10453):707–24. 10.1016/S0140-6736(24)01136-X. 39096925 10.1016/S0140-6736(24)01136-X

[3] Molnar T, Lehoczki A, Fekete M, Varnai R, Zavori L, Erdo-Bonyar S, et al. Mitochondrial dysfunction in long COVID: mechanisms, consequences, and potential therapeutic approaches. GeroScience. 2024;46(5):5267–86. 10.1007/s11357-024-01165-5. 38668888 10.1007/s11357-024-01165-5 PMC11336094

[4] Guarnieri JW, Dybas JM, Fazelinia H, Kim MS, Frere J, Zhang Y, et al. Core mitochondrial genes are down-regulated during SARS-CoV‑2 infection of rodent and human hosts. Sci Transl Med. 2023;15(708):eabq1533. 10.1126/scitranslmed.abq1533. 37556555 10.1126/scitranslmed.abq1533 PMC11624572

[5] Appelman B, Charlton BT, Goulding RP, Kerkhoff TJ, Breedveld EA, Noort W, et al. Muscle abnormalities worsen after post-exertional malaise in long COVID. Nat Commun. 2024;15(1):17. 10.1038/s41467-023-44432-3. 38177128 10.1038/s41467-023-44432-3 PMC10766651

[6] Capone F, Salati S, Vincenzi F, Liberti M, Aicardi G, Apollonio F, et al. Pulsed electromagnetic fields: a novel attractive therapeutic opportunity for neuroprotection after acute cerebral ischemia. Neuromodulation. 2022;25(8):1240–7. 10.1111/ner.13489. 34480781 10.1111/ner.13489

[7] Yang C, Xu L, Liao F, Liao C, Zhao Y, Chen Y, Yu Q, et al. Pulsed electromagnetic fields regulate metabolic reprogramming and mitochondrial fission in endothelial cells for angiogenesis. Sci Rep. 2024;14(1):19027. 10.1038/s41598-024-69862-x. 39152229 10.1038/s41598-024-69862-x PMC11329790

[8] Wagner B, Steiner M, Markovic L, Crevenna R. Successful application of pulsed electromagnetic fields in a patient with post-COVID-19 fatigue: a case report. Wien Med Wochenschr. 2022;172(9):227–32. 10.1007/s10354-021-00901-2. 35006516 10.1007/s10354-021-00901-2 PMC8743351

[9] Machado FVC, Meys R, Delbressine JM, Vaes AW, Goërtz YMJ, van Herck M, et al. Construct validity of the post-COVID-19 functional status scale in adult subjects with COVID-19. Health Qual Life Outcomes. 2021;19(1):40. 10.1186/s12955-021-01691-2. 33536042 10.1186/s12955-021-01691-2 PMC7856622

[10] Papimi™ official homepage https://www.papimi.com/. Accessed 25 Nov 2024.

[11] Holland AE, Spruit MA, Troosters T, Puhan MA, Pepin V, Saey D, McCormack MC, et al. An official European Respiratory Society/American Thoracic Society technical standard: field walking tests in chronic respiratory disease. Eur Respir J. 2014;44(6):1428–46. 10.1183/09031936.00150314. 25359355 10.1183/09031936.00150314

[12] Torres-Castro R, Núñez-Cortés R, Larrateguy S, Alsina-Restoy X, Barberà JA, Gimeno-Santos E, et al. Assessment of exercise capacity in post-COVID-19 patients: how is the appropriate test chosen? Life. 2023;13(3):621. 10.3390/life13030621. 36983777 10.3390/life13030621 PMC10054514

[13] Sevillano-Castaño A, Peroy-Badal R, Torres-Castro R, Gimeno-Santos E, García Fernández P, Garcia Vila C, et al. Is there a learning effect on 1‑min sit-to-stand test in post-COVID-19 patients? ERJ Open Res. 2022;8(3):189–2022. 10.1183/23120541.00189-2022. 36171984 10.1183/23120541.00189-2022 PMC9511157

[14] Rostamzadeh S, Allafasghari A, Allafasghari A, Abouhossein A. Handgrip strength as a prognostic factor for COVID-19 mortality among older adult patients admitted to the intensive care unit (ICU): a comparison Alpha (B.1.1.7) and Delta (B.1.617.2) variants. Sci Rep. 2024;14(1):19927. 10.1038/s41598-024-71034-w. 39198687 10.1038/s41598-024-71034-w PMC11358457

[15] Mathiowetz V. Grip and pinch strength measurements. In: Amundsen LR, editor. Muscle strength testing instrumented and noninstrumented systems. New York: Churchill Livingstone; 1990. pp. 163–77.

[16] Shuman-Paretsky MJ, Belser-Ehrlich J, Holtzer R. Psychometric properties of the brief fatigue inventory in community-dwelling older adults. Arch Phys Med Rehabil. 2014;95(8):1533–9. 10.1016/j.apmr.2014.03.026. 24742938 10.1016/j.apmr.2014.03.026 PMC4124821

[17] Radbruch L, Sabatowski R, Elsner F, Everts J, Mendoza T, Cleeland C. Validation of the German version of the brief fatigue inventory. J Pain Symptom Manage. 2003;25(5):449–58. 10.1016/s0885-3924(03)00073-3. 12727043 10.1016/s0885-3924(03)00073-3

[18] Tacchini-Jacquier N, Monnay S, Coquoz N, Bonvin E, Verloo H. Patient-reported experiences of persistent post-COVID-19 conditions after hospital discharge during the second and third waves of the pandemic in Switzerland: cross-sectional questionnaire study. JMIR Public Health Surveill. 2024;10:e47465. 10.2196/47465. 39197160 10.2196/47465 PMC11391158

[19] Smets EM, Garssen B, Bonke B, De Haes JC. The multidimensional fatigue inventory (MFI) psychometric qualities of an instrument to assess fatigue. J Psychosom Res. 1995;39(3):315–25. 10.1016/0022-3999(94)00125-o. 7636775 10.1016/0022-3999(94)00125-o

[20] Freynhagen R, Baron R, Gockel U, Tölle TR. painDETECT: a new screening questionnaire to identify neuropathic components in patients with back pain. Curr Med Res Opin. 2006;22(10):1911–20. 10.1185/030079906X132488. 17022849 10.1185/030079906X132488

[21] Bjelland I, Dahl AA, Haug TT, Neckelmann D. The validity of the hospital anxiety and depression scale. An updated literature review. J Psychosom Res. 2002;52(2):69–77. 10.1016/s0022-3999(01)00296-3. 11832252 10.1016/s0022-3999(01)00296-3

[22] Fabbri M, Beracci A, Martoni M, Meneo D, Tonetti L, Natale V. Measuring subjective sleep quality: a review. Int J Environ Res Public Health. 2021;18(3):1082. 10.3390/ijerph18031082. 33530453 10.3390/ijerph18031082 PMC7908437

[23] Lins L, Carvalho FM. SF-36 total score as a single measure of health-related quality of life: scoping review. SAGE Open Med. 2016;4:2050312116671725. 10.1177/2050312116671725. 27757230 10.1177/2050312116671725 PMC5052926

[24] Bullinger M. German translation and psychometric testing of the SF-36 health survey: preliminary results from the IQOLA project. International quality of life assessment. Soc Sci Med. 1995;41:1359–66. 8560303 10.1016/0277-9536(95)00115-n

[25] van den Berg TI, Elders LA, de Zwart BC, Burdorf A. The effects of work-related and individual factors on the work ability index: a systematic review. Occup Environ Med. 2009;66(4):211–20. 10.1136/oem.2008.039883. 19017690 10.1136/oem.2008.039883

[26] van Buuren S, Groothuis-Oudshoorn K. mice: multivariate imputation by chained equations in R. J Stat Softw. 2011;45(3):1–67. 10.18637/jss.v045.i03.

[27] Ross CL, Zhou Y, McCall CE, Soker S, Criswell TL. The use of pulsed electromagnetic field to modulate inflammation and improve tissue regeneration: a review. Bioelectricity. 2019;1(4):247–59. 10.1089/bioe.2019.0026. 34471827 10.1089/bioe.2019.0026 PMC8370292

[28] Yang X, He H, Ye W, Perry TA, He C. Effects of pulsed electromagnetic field therapy on pain, stiffness, physical function, and quality of life in patients with osteoarthritis: a systematic review and meta-analysis of randomized placebo-controlled trials. Phys Ther. 2020;100(7):1118–31. 10.1093/ptj/pzaa054. 32251502 10.1093/ptj/pzaa054

[29] Friscia M, Abbate V, De Fazio GR, Sani L, Spinelli R, et al. Pulsed electromagnetic fields (PEMF) as a valid tool in orthognathic surgery to reduce post-operative pain and swelling: a prospective study. Oral Maxillofac Surg. 2024;28(3):1287–94. 10.1007/s10006-024-01256-9. 38698248 10.1007/s10006-024-01256-9 PMC11330404

[30] Factor S, Druckmann I, Kazum E, Atlan F, Tordjman D, Rosenblatt Y, et al. A novel pulsed electromagnetic field device as an adjunct therapy to surgical treatment of distal radius fractures: a prospective, double-blind, sham-controlled, randomized pilot study. Arch Orthop Trauma Surg. 2024;144(1):543–50. 10.1007/s00402-023-05117-0. 37971511 10.1007/s00402-023-05117-0

[31] Hug K, Röösli M. Therapeutic effects of whole-body devices applying pulsed electromagnetic fields (PEMF): a systematic literature review. Bioelectromagnetics. 2012;33(2):95–105. 10.1002/bem.20703. 21938735 10.1002/bem.20703

[32] Markovic L, Wagner B, Crevenna R. Effects of pulsed electromagnetic field therapy on outcomes associated with osteoarthritis: a systematic review of systematic reviews. Wien Klin Wochenschr. 2022;134(11):425–33. 10.1007/s00508-022-02020-3. 35362792 10.1007/s00508-022-02020-3 PMC9213303

[33] Kull P, Keilani M, Remer F, Crevenna R. Efficacy of pulsed electromagnetic field therapy on pain and physical function in patients with non-specific low back pain: a systematic review. Wien Med Wochenschr. 2023; 10.1007/s10354-023-01025-5. 37999784 10.1007/s10354-023-01025-5 PMC11775040

[34] Mostert S, Kesselring J. Effect of pulsed magnetic field therapy on the level of fatigue in patients with multiple sclerosis—a randomized controlled trial. Mult Scler. 2005;11(3):302–5. 10.1191/1352458505ms1156oa. 15957511 10.1191/1352458505ms1156oa

[35] Granja-Domínguez A, Hochsprung A, Luque-Moreno C, Magni E, Escudero-Uribe S, Heredia-Camacho B, et al. Effects of pulsed electromagnetic field therapy on fatigue, walking performance, depression, and quality of life in adults with multiple sclerosis: a randomized placebo-controlled trial. Braz J Phys Ther. 2022;26(5):100449. 10.1016/j.bjpt.2022.100449. 36283240 10.1016/j.bjpt.2022.100449 PMC9594115

[36] Bodrova RA, Kuchumova TV, Zakamyrdina AD, Yunusova ER, Fadeev GY. Efficacy of low-frequency magnetic therapy in patients with COVID-19 pneumonia. Vopr Kurortol Fizioter Lech Fiz Kult. 2020;97(6):11–6. 10.17116/kurort20209706111. 33307658 10.17116/kurort20209706111

[37] Zhang JX, Zhang JJ. Case report of improvement in long-COVID symptoms in an air force medic treated with transcranial magnetic stimulation using electro-magnetic brain pulse technique. Mil Med. 2023;188(11):e3711–e5. 10.1093/milmed/usad182. 37267198 10.1093/milmed/usad182

[38] Schaefer LV, Bittmann FN. Case report: individualized pulsed electromagnetic field therapy in a long COVID patient using the adaptive force as biomarker. Front Med. 2023;9:879971. 10.3389/fmed.2022.879971. 10.3389/fmed.2022.879971 PMC9874300 36714125

[39] Chen TH, Chang CJ, Hung PH. Possible pathogenesis and prevention of long COVID: SARS-CoV-2-induced mitochondrial disorder. Int J Mol Sci. 2023;24(9):8034. 10.3390/ijms24098034. 37175745 10.3390/ijms24098034 PMC10179190

[40] Moine E, Molinier V, Castanyer A, Calvat A, Coste G, Vernet A, et al. Safety and efficacy of pulmonary rehabilitation for long COVID patients experiencing long-lasting symptoms. Int J Environ Res Public Health. 2024;21(2):242. 10.3390/ijerph21020242. 38397731 10.3390/ijerph21020242 PMC10888408

[41] Pfoser-Poschacher V, Keilani M, Steiner M, Schmeckenbecher J, Zwick RH, Crevenna R. Feasibility and acceptance of transdermal auricular vagus nerve stimulation using a TENS device in females suffering from long COVID fatigue. Wien Klin Wochenschr. 2025; 10.1007/s00508-025-02501-1. 39969545 10.1007/s00508-025-02501-1 PMC12534353

[42] Rabady S, Hoffmann K, Aigner M, Altenberger J, Brose M, Costa U, et al. Leitlinie S1 für das Management postviraler Zustände am Beispiel Post-COVID-19. Wien Klin Wochenschr. 2023;135(4):525–98. 10.1007/s00508-023-02242-z. 37555900 10.1007/s00508-023-02242-z PMC10504206

